# Exploring the Mechanism of Luteolin in Protecting Chickens from Ammonia Poisoning Based on Proteomic Technology

**DOI:** 10.3390/metabo15050326

**Published:** 2025-05-14

**Authors:** Yu Jin, Azi Shama, Haojinming Tang, Ting Zhao, Xinyu Zhang, Falong Yang, Dechun Chen

**Affiliations:** 1 College of Animal and Veterinary Sciences, Southwest Minzu University, Chengdu 610041, China; 202131304036@stu.swun.edu.cn (Y.J.); 202131307029@stu.swun.edu.cn (A.S.); 240906032007@stu.swun.edu.cn (T.Z.); 230952002001@stu.swun.edu.cn (H.T.); 240952002046@stu.swun.edu.cn (X.Z.); falong.yang@swun.edu.cn (F.Y.); 2 Key Laboratory of Animal Medicine in Sichuan Province, Southwest Minzu University, Chengdu 610041, China

**Keywords:** ammonia, chicken, luteolin, anti-inflammatory, apoptosis, proteomics

## Abstract

Background: Ammonia (NH_3_), a harmful gas, reduces livestock productivity, threatens their health, and causes economic losses. Luteolin (Lut), an anti-inflammatory flavonoid, may counteract these effects. Methods: Our study explored luteolin’s protective mechanisms on chicken splenic lymphocytes under ammonia stress using a simulation model and four-dimensional fast data-independent acquisition (4D-FastDIA) proteomics. We identified 316 proteins, with 69 related to ammonia’s negative effects and 247 to Lut’s protection. Thirty differentially expressed proteins (DEPs) were common to both groups, with 27 showing counter-regulation with Lut. Results: Gene Ontology (GO) analysis showed DEPs enriched in molecular responses to interferons and the negative regulation of immune responses, mainly located extracellularly. Molecular function analysis revealed DEPs in antigen binding and synthase activity. Kyoto Encyclopedia of Genes and Genomes (KEGG) analysis linked DEPs to pathways like estrogen signaling, NOD-like receptor signaling, cytokine–cytokine receptor interaction, and JAK-STAT signaling. The quantitative real-time polymerase chain reaction (qRT-PCR) results indicated that the mRNA levels of Interferon Alpha and Beta Receptor subunit 2 (IFNAR2) and Signal Transducer and Activator of Transcription 1 (STAT1) were trending downward. This observation was in strong agreement with the downregulation noted in the proteomics analysis. Conclusions: Lut’s protective role against ammonia’s adverse effects on chicken splenic lymphocytes is linked to the modulation of key signaling pathways, offering insights for further research on treating ammonia exposure with Lut.

## 1. Introduction

Ammonia (NH_3_), a toxic environmental pollutant in poultry production systems [1], is primarily generated through enzymatic hydrolysis of feed proteins in the digestive tract [2] and microbial degradation of nitrogenous compounds in excreta [3]. Suboptimal ventilation and delayed waste removal in confined coops elevate ambient NH_3_ concentrations, impairing broiler productivity and inducing pathophysiological damage. Notably, chronic NH_3_ exposure triggers splenic dysfunction via pyroptosis [4] and apoptosis [5] of splenocytes, ultimately reducing growth performance [6] and incurring substantial economic losses to the poultry industry.

Luteolin (Lut), a naturally occurring flavonoid compound widely distributed across various plants [7], has garnered significant attention due to its diverse pharmacological activities, particularly its notable efficacy in the realm of anti-inflammatory treatment and antioxidant properties [8,9]. Previous studies had confirmed that Lut can effectively alleviate inflammatory responses [10] and shield the body from NLRP3-mediated inflammatory diseases [11]. In the specific domain of preventing ammonia poisoning in chickens, current research revealed that Lut not only reduced lymphocyte autophagy and apoptosis induced by ammonia exposure [12] but also significantly mitigated inflammatory reactions and pyroptosis levels [13]. Consequently, Lut has demonstrated its therapeutic potential across a broad spectrum of pharmacological applications, especially in alleviating inflammation and cellular damage caused by ammonia. This offers innovative scientific insights and approaches for combating ammonia toxicity within the poultry sector.

Proteomics plays a critical role in toxin avoidance and animal husbandry, achieving significant research advancements [14]. In toxin prevention, proteomics has been deployed to study the effects of toxins on living organisms. The hepatotoxicity of sodium diclofenac in broiler chickens may have been mediated through the induction of hepatocyte apoptosis and interference with retinol and purine metabolism [15]. In the field of ammonia poisoning research, studies utilizing proteomic techniques revealed how NH_3_ could trigger thymic injury by activating immune responses, disrupting metabolic processes, and inducing apoptosis [16]. Within the livestock industry, proteomic technology held substantial application value. Proteomic analyses had demonstrated that fermented diets had enhanced the insulin/AKT/mTORC1 signaling axis, and the Foxo1/MAFbx pathway was modulated, affecting ribosomal and myogenic proteins and enhancing pork quality and pectoralis major development [17]. Additionally, in this study, we employed proteomics to investigate the molecular protective mechanisms of Lut against ammonia poisoning in chickens. Our findings not only address a research gap but also highlight the potential of Lut as a therapeutic agent in mitigating the harmful effects of ammonia. By elucidating the molecular mechanisms through which Lut exerts its protective effects, this study contributes to the development of strategies for environmental health and pollution control.

## 2. Experimental Methods

### 2.1. Ethical Considerations

The animal study protocol was approved by the Academic Ethics and Morality Committee of Southwest Minzu University (SWUN-2022MR0056, March 2022).

### 2.2. Primary Cell Culture and Construction of the Lut-Protected Ammonia Poisoning Injury Model

Spleens were collected from 45-day-old Ross 308 broilers (*n* = 8/group, mixed-sex cohort) raised under controlled conditions (22 ± 1 °C, 55% humidity, and a 16:8 light/dark cycle) with ad libitum access to commercial a starter-grower diet (ME 3000 kcal/kg, CP 21.5%; Zhengbang Group, Nanchang, China) and acidified water (pH 2.5–3.0). Animals were confirmed pathogen-free through weekly ELISA screening (NDV/IBV/CAV). Cardiac blood was collected from anesthetized broilers (sodium pentobarbital 30–40 mg/kg and IV via the wing vein) via standardized thoracic puncture prior to spleen excision. Following the chickens’ humane euthanasia, their cardiac blood was gathered. Cells were cultured in the RPMI-1640 medium (Bio-O-Novo) supplemented with 10% FBS (Bioint, CA, USA) and 1% penicillin/streptomycin (Hyclone) at 1.0 × 10^7^ cells/mL under 5% CO_2_/37 °C. For ammonia exposure modeling, 1 mmol/L ammonium chloride (NH_4_Cl) [5] (Zhongtian Fine Chemicals, Jinan, China, purity ≥ 99.5%) was applied post 12 h Lut pretreatment (0.5 μg/mL [12]). Poly-L-lysine-coated 96-well plates received 100 μL of cell suspension per well, with the Lut group receiving 100 μL of Lut (3 h post-seeding) versus an equal volume of the fresh medium in controls. After the 12 h NH_4_Cl challenge (all groups except the control), cells underwent 12 h of incubation prior to PBS washing, were flash-frozen in liquid nitrogen, and then stored at −80 °C for proteomics.

### 2.3. Protein Isolation and Proteolytic Processing

After storage at −80 °C, samples were lysed in an SDS-enriched buffer (1% SDS and 1% protease/phosphatase inhibitors) and sonicated for cellular disruption. Following centrifugation (12,000× *g* at 4 °C for 10 min), supernatants were collected for protein quantification using a bicinchoninic acid (BCA) assay. Equalized samples underwent acetone precipitation (1:4 vol/vol chilled acetone at −20 °C for 2 h). After centrifugation (4500× *g* for 5 min), pellets were washed twice with chilled acetone, air-dried, and solubilized in 200 mM triethylammonium bicarbonate (TEAB) with brief sonication. For enzymatic digestion, trypsin (1:50 enzyme/protein) was added after disulfide bond reduction with 5 mM dithiothreitol (DTT) (56 °C, 30 min). Alkylation was performed using 11 mM iodoacetamide (IAA) (at room temperature in the dark for 15 min) to stabilize cysteine residues prior to analysis.

### 2.4. Utilizing Liquid Chromatography and Tandem Mass Spectrometry for Quantitative Analysis

Peptides were dissolved in solvent A, the high-performance liquid chromatography (HPLC) mobile phase, and were separated using the EASY-nLC 1200 HPLC system. Solvent A was an aqueous solution containing 0.1% formic acid and 2% acetonitrile, and solvent B was an aqueous solution containing 0.1% formic acid and 90% acetonitrile. The HPLC gradient program was carefully set up as follows: a linear gradient from 6% to 22% B for the first 22.5 min, an increase to 34% B for 26.5 min, a to 80% B for 28.5 min, a further increase to 80% B for 28.5 min, and held at 80% B for a maximum of 30 min; the flow rate was maintained at 700 nL/min. After separation, the peptides were first introduced into an NSI ion source for ionization. They were then transferred to an Orbitrap Exploris 480 mass spectrometer (Thermo Fisher Scientific, Waltham, MA, USA) for comprehensive analysis. The ion source was operated at 2300 V. A high-resolution Orbitrap detector was used to identify peptide precursor ions and their corresponding fragments. The mass spectrometry 1 (MS1) scan range was set between 350 and 1400 *m*/*z* with a resolution of 60,000, while the MS2 scan method was performed at 120 *m*/*z* with a resolution of 15,000. For data acquisition, a data-independent acquisition (DIA) strategy was employed. Peptide ions were fragmented within multiple *m*/*z* windows at 27% collision energy in the HCD cell prior to MS2 analysis. To optimize the performance of the mass spectrometer, the automatic gain control (AGC) target was set to 1E6, and the maximum injection time was limited to 22 ms.

### 2.5. Database Searching

For the execution of this experiment, DIA data were interrogated utilizing the Pulsar search engine integrated within Spectronaught version 17, employing the software’s default parameters for the analysis. The reference database employed was Gallus_gallus_9031_PR_20230328.fasta, encompassing 43,710 sequences, with an accompanying decoy database to ascertain the false discovery rate (FDR) attributable to random alignments. The settings permitted up to 2 missed cleavage sites. Carbamidomethylation of cysteine (Carbamidomethyl, C) was defined as a constant modification, while variable modifications included methionine oxidation and acetylation at the protein’s N-terminus. The false discovery rate (FDR) cutoffs for protein, peptide, and peptide-spectrum match (PSM) identification were all maintained at 1%. To ensure the delivery of high-fidelity analytical outcomes, subsequent filtration of the search results was imperative. The criteria for filtering the identification outcomes were meticulously defined as follows: both precursor and protein FDR were maintained at 1%; identified proteins were required to harbor at least one unique peptide; the normalized intensity of proteins was derived by employing the MSstats R package, ensuring a robust quantification of proteomic data.

### 2.6. Data Quality Control and Quantitative Analysis

Within the domain of data quality control, our study meticulously scrutinized various parameters, including peptide lengths, quantities, protein coverage, and molecular weight distributions. In the quantitative analysis segment, the research concentrated on two fundamental aspects: reproducibility analysis and intensity value analysis. By upholding rigorous quality control measures, we guaranteed the accuracy and reliability of the proteomics data, thus laying a solid foundation for in-depth data analysis and comprehensive biological interpretation.

### 2.7. Bioinformatics Analysis

#### 2.7.1. Functional Annotation of Differentially Expressed Proteins (DEPs)

To comprehensively elucidate the functional attributes of diverse proteins, a systematic annotation strategy was employed. Initially, the proteins of interest were annotated using the eggnog-mapper software (v2.1.6), leveraging the EggNOG database to facilitate Gene Ontology (GO) analysis and categorize them into clusters of orthologous groups of proteins (COGs), thereby enabling cross-species functional conservation analysis. Subsequently, proteins were annotated with structural domains using project-specific data, the Pfam database for domain identification, and the PfamScan tool to provide mechanistic insights into protein architecture. Moreover, to contextualize biological roles, the pathways of proteins were identified by consulting the Kyoto Encyclopedia of Genes and Genomes (KEGG) database. To further refine the annotations, the identified proteins underwent a BLAST (v2.13.0) comparison (blastp, using an *e*-value threshold of ≤0.0001). In each instance, the annotation was prioritized based on the alignment with the highest scoring match to ensure reliability. Ultimately, this multi-step annotation strategy enabled a comprehensive understanding of protein functions and their biological roles.

#### 2.7.2. Functional Classification and Enrichment Analysis

GO analysis is a pivotal bioinformatics approach that systematically correlates genes and their products, notably proteins, with a spectrum of functional annotations, thereby furnishing a rich dataset for statistical inference. In the context of our study, we employed the eggnog-mapper software, predicated on the EggNOG database, to ascertain GO identifiers (IDs) for each protein under investigation. Subsequently, the proteins were meticulously categorized and annotated based on their roles within the cell, their molecular functions, and their involvement in biological processes. To enhance our understanding of the biological significance, we conducted a GO enrichment analysis. This analysis employed Fisher’s exact test to assess the statistical enrichment of DEPs in comparison with the broader protein dataset identified in our research. A significance threshold was set at a P-value of less than 0.05 to indicate statistically significant enrichment.

#### 2.7.3. Enrichment-Based Clustering

After the completion of GO classification and enrichment analysis for DEPs across multiple comparative groups, we proceeded with clustering analysis to elucidate the functional similarities and disparities among these proteins. This approach was utilized to uncover potential correlations and distinctions within specific functional KEGG pathways. Initially, we compiled a comprehensive dataset encompassing the functional categorization specifics and the associated enrichment P-values for the protein groups. Subsequently, we focused on functional classifications that exhibited significant enrichment. (*p* < 0.05) was observed in at least one of the protein groups. Subsequently, the *p*-value dataset underwent logarithmic scaling with base −Log10 to standardize the distribution of the data. This transformed dataset was then analyzed using one-sided hierarchical clustering. The clustering outcomes were graphically represented through the heatmap function from the R package Complex Heatmap, generating a heatmap that visually encapsulated the clustering relationships.

#### 2.7.4. Protein–Protein Interaction (PPI) Network

DEPs were ascertained from the comparative groups by applying a 1.5-fold change criterion for sequence identification and were subjected to analysis against the STRING database, which models protein–protein interaction networks. Interactions possessing a confidence score surpassing 0.7, indicating high confidence, were extracted to elucidate the interactions among differential proteins. Following this, the R package “visNetwork” was employed to graphically represent the network of differential protein interactions, providing a visual synthesis of the data.

### 2.8. Comparative Analysis of mRNA Expression Levels

Trizol reagent from TaKaRa (Otsu, Japan), was used to manually isolate lymphocyte RNA. The isolated RNA was then synthesized following the procedure provided by ABclonal Technology Co. Ltd., Wuhan, China (Table 1). Primers targeting genes such as β-actin, Interferon Regulatory Factor 7 (IRF-7), Interferon-alpha (IFN-α), Interferon Alpha and Beta Receptor subunit (IFNAR2), Cyclic AMP-responsive Element-binding Protein (CRE), Cysteinyl Aspartate Specific Protease 9 (Caspase-9), Signal Transducer and Activator of Transcription 1 (STAT1), Apoptosis Inhibitor 5 (API-5), and Suppressor of Cytokine Signaling 3 (SOCS-3) are available from Sangon Co. Biotech Ltd. (Shanghai, China) and were obtained. The quantitative real-time polymerase chain reaction (qRT-PCR) experiment was conducted using the Roche LightCycler^®^ 480 II detection system (Roche, Basel, Switzerland). This instrument is manufactured in Switzerland. The 2^−ΔΔCt^ method was employed. Subsequently, the relative expression levels of mRNAs were evaluated.

### 2.9. Analytical Statistics

Data analysis was conducted using GraphPad Prism (version 9.3.1). For comparisons among the three groups, statistical significance was determined by one-way ANOVA followed by Tukey’s post hoc test for multiple comparisons. The results are presented as columns, with an asterisk (*) indicating significant differences (*p* < 0.05). The significance levels are categorized as follows: * for *p* < 0.05, ** for *p* < 0.01, *** for *p* < 0.001, and **** for *p* < 0.0001. For comparisons between two groups, unpaired Student’s *t*-test was applied using identical asterisk-based significance labeling. All data met normality assumptions as verified by Shapiro–Wilk tests.

## 3. Results and Analysis

### 3.1. Analysis of Data Quality Assurance

**Figure 1:**
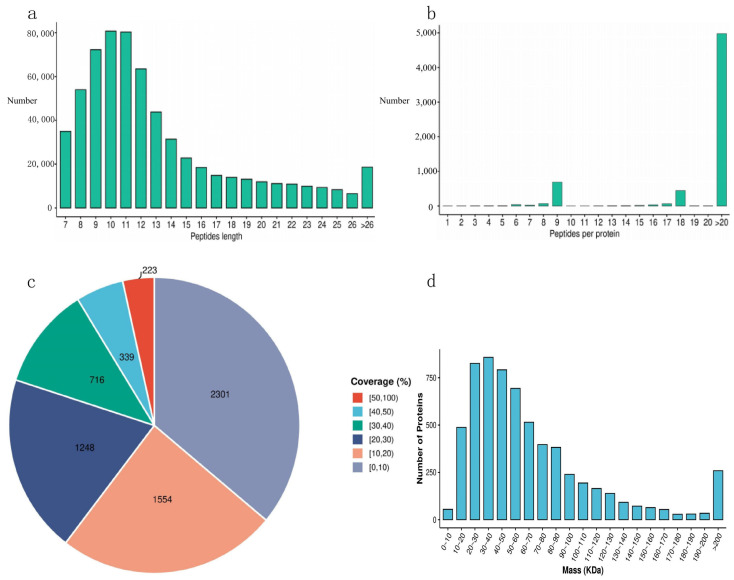
Proteomics data quality control. (**a**) Peptide length distribution plot. The horizontal axis represents the length of the peptides, and the vertical axis represents the number of peptides. (**b**) Peptide count distribution plot. The horizontal axis represents the number of peptides per protein, and the vertical axis represents the number of proteins. (**c**) Protein coverage distribution plot. Different colored areas in the plot represent different protein coverage intervals, with the numbers in parentheses indicating the number of proteins in each interval and the percentages showing the proportion of proteins in that interval relative to the total number of proteins. (**d**) Protein molecular weight distribution plot. The horizontal axis represents the molecular weight, and the vertical axis represents the number of proteins.

The assessment of data quality is depicted in Figure 1, encompassing the examination of peptide length dispersion, the distribution of peptide counts, the spread of protein coverage, and the distribution of protein molecular weights. As depicted in Figure 1a, the majority of peptides were found within the 7 to 20 amino acid range, with a notable peak at 10 to 11 amino acids. This distribution corresponded to the expected patterns from proteolytic cleavage and mass spectrometric dissociation, thereby meeting our quality control benchmarks. Figure 1b shows that the majority of proteins are associated with two or more peptides, aligning with our quality control specifications. Figure 1c revealed that the majority of proteins had a coverage below 30%, with most proteins falling within the 0–10% range. Given the shotgun strategy employed in mass spectrometry analysis, there was a natural inclination towards the detection of more abundant peptides. Consequently, protein coverage was positively correlated with abundance within the sample, and the distribution of protein coverage also met our quality control standards. Figure 1d demonstrates that the molecular weights of the identified proteins are evenly spread across all ranges, with a particular concentration in the 30 to 40 kDa range. This distribution indicated that no proteins within specific molecular weight brackets were selectively lost during the experimental procedure, adhering to our quality control standards.

### 3.2. Protein Quantitative Analysis

**Figure 2:**
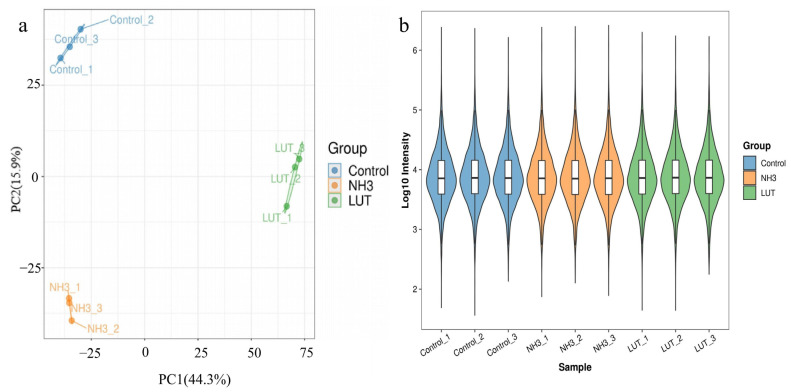
Quantitative analysis in proteomics. (**a**) Principal component analysis (PCA) plot. The horizontal and vertical axes display the explained variance of PC1 and PC2, with higher values indicating greater explained variance. The degree of clustering within a group represents the quality of replicate sampling within that group. (**b**) Intensity violin distribution plot. The horizontal axis represents the sample names, and the vertical axis represents the intensity values after Log10 transformation. The color of the violin represents different groups. Inside the violin plot is a boxplot, where the box represents the middle 50% distribution interval of the data for that group, and the outer part is a kernel density plot. The larger the area of the regional shape, the greater the probability of the corresponding value distribution. Horizontal comparison can roughly show the dispersion of data distribution within and between groups.

Protein quantification analysis, a pivotal element of our research, comprised two principal components: reproducibility and intensity value analysis (Figure 2). To evaluate the statistical concordance of the quantification outcomes among replicates, we utilized principal component analysis (PCA), a widely recognized statistical tool for assessing reproducibility. The PCA was performed using the relative quantification data from all samples, and the outcomes were graphically represented in a PCA plot. Figure 2a demonstrates that the replicate samples within each group are inclined to cluster closely, which is indicative of robust reproducibility. Additionally, to delve into the distribution and heterogeneity of protein intensity values across various samples, we extracted these values and employed violin plots for their visualization. Figure 2b reveals that the sample means are aligned along a consistent horizontal line, a testament to the superior quality of the samples. Consequently, based on these findings, the reproducibility and intensity value analyses conducted in this study have successfully adhered to the predetermined standards.

### 3.3. Protein Functional Annotation

**Figure 3:**
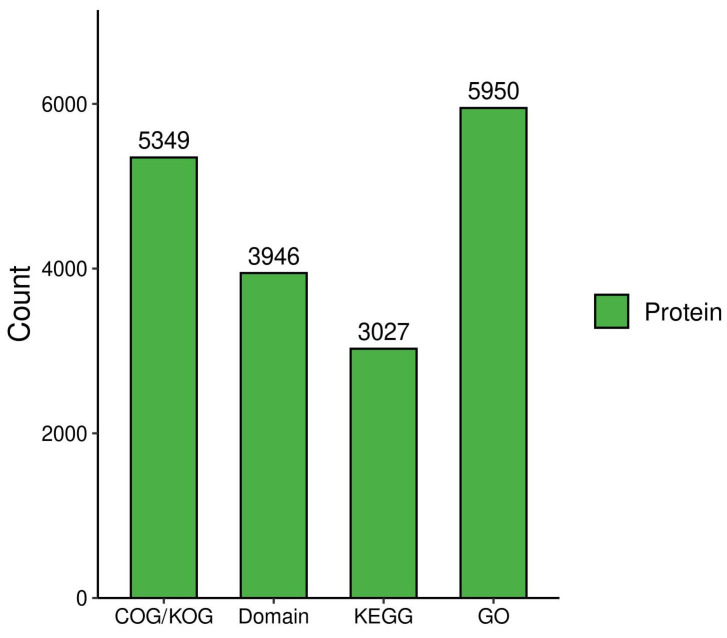
Protein function annotation. Bar chart illustrating the quantitative distribution of proteins across four major functional databases: COG/KOG (5349 proteins), domain analysis (3946 proteins), KEGG (3027 proteins), and GO (5950 proteins). The Y-axis represents protein counts (scale: 0–6000).

To achieve a holistic view of the functional attributes of various proteins, we performed an exhaustive functional characterization of the proteins detected, with the findings depicted in Figure 3. We annotated the protein functions across four dimensions: COG/KOG functional classification, protein domains, KEGG pathways, and GO. The number of protein functions annotated by each method was 5349, 3946, 3027, and 5950, respectively. These data provided us with a wealth of information that enables a more precise comparison.

### 3.4. The Specific Number of DEPs

**Figure 4:**
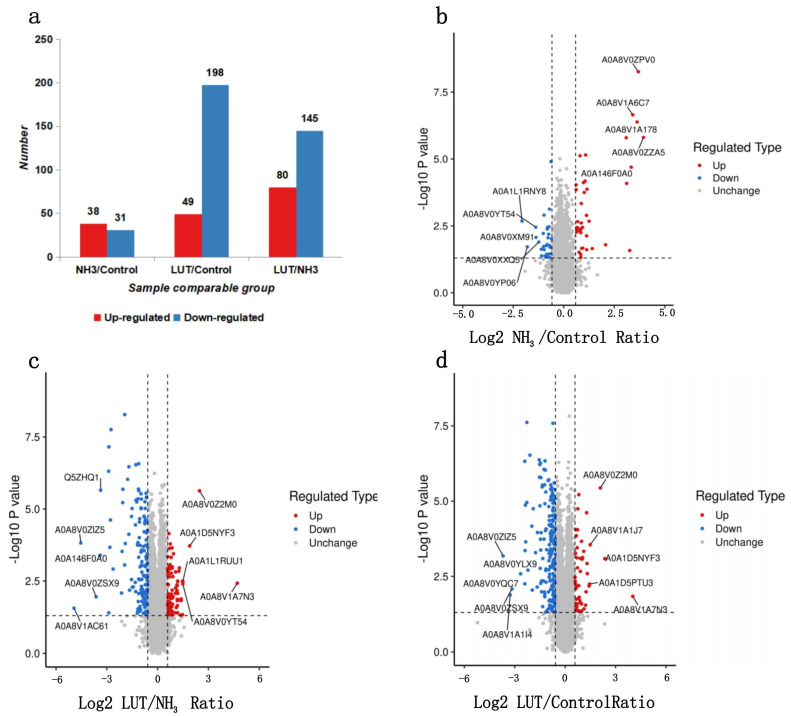
Number of DEPs between groups and volcano plot. (**a**) Bar graph of the number of DEPs between groups. (**b**) Volcano plot of differential proteins between the NH_3_ group and the control group. (**c**) Volcano plot of differential proteins between the Lut group and the NH_3_ group. (**d**) Volcano plot of differential proteins between the Lut group and the control group. In Figure 4 (**b**–**d**), the horizontal axis represents the Log2-transformed fold change (Ratio) values of differential expression between comparison groups; the vertical axis represents the −Log10-transformed *p* values from the *T*-test; red dots indicate significantly upregulated proteins; blue dots indicate significantly downregulated proteins, and gray indicates no significant difference. The top 5 upregulated and downregulated proteins (ranked by the absolute value of the Log2 ratio from largest to smallest) are also labeled on the plots.

Following a meticulous series of procedures that included protein extraction, enzymatic digestion, and liquid chromatography separation, stringent criteria were applied, with the precursor and protein FDR meticulously set to 1%. Under these conditions, mass spectrometry analysis had successfully identified 54,441 peptides, encompassing 27,649 unique peptides. Furthermore, this study had delineated a total of 6381 proteins, with 6380 of these being quantifiable. To ascertain significant alterations in protein expression, a cutoff exceeding a 1.5-fold variation for upregulation and less than 1/1.5-fold change for downregulation was employed, augmented with a significance level of *p* < 0.05. As shown in Figure 4a, screening yielded 541 DEPs across three comparisons. Comparative analysis between the NH_3_ treatment group and the control group (NH_3_ vs. Control) revealed 69 DEPs, with 38 being significantly upregulated and 31 significantly downregulated. When contrasting the NH_3_ treatment group with the Lut treatment group (NH_3_ vs. Lut), 247 DEPs were identified, consisting of 49 proteins that were upregulated and 198 that were downregulated. In the analysis contrasting the Lut treatment group with the control group (Lut vs. Control), a total of 225 DEPs were detected, including 80 that were upregulated and 145 that were downregulated. Notably, 30 DEPs were shared between the NH_3_ vs. Control and Lut vs. Control comparisons. Strikingly, within this subset, 27 proteins (90%) exhibited expression patterns counter-regulated by Lut treatment.

The comparative analysis of protein expression levels had been graphically represented in the volcano plots of Figure 4b–d. Figure 4b shows the most significant alterations in the NH_3_ vs. Control group, where the top five upregulated proteins were identified as Keratin 12, Keratin 14, an IF rod domain-containing protein, and Keratin 7. Conversely, the top five downregulated proteins in this comparison were Histone H2A, TELO2 interacting protein 1, an Ig-like domain-containing protein, DExD/H-box helicase 60, and an H15 domain-containing protein. Figure 4c presents the NH_3_ vs. Lut group, highlighting the top five upregulated proteins, which include Granulysin, fatty acid-binding protein 4, an uncharacterized protein, TLE family member 5, and TELO2-interacting protein 1. The top five downregulated proteins in this group were small ubiquitin-related modifier 3, glutathione hydrolase, Keratin 7, URI1 prefoldin-like chaperone, and Keratin 5. Lastly, Figure 4d illustrates the Lut vs. Control group, with the top five upregulated proteins being Granulysin, Jun proto-onco, fatty acid-binding protein 4, Tumor Necrosis Factor (TNF) receptor superfamily member 1B, and TLE family member 5. The top five downregulated proteins in this comparison were glutathione hydrolase, creatine kinase, Stem-loop binding protein, URI1 prefoldin-like chaperone, and ubiquitin-conjugating enzyme E2.

### 3.5. Results of GO Functional Classification and Enrichment Analysis of DEPs

**Figure 5:**
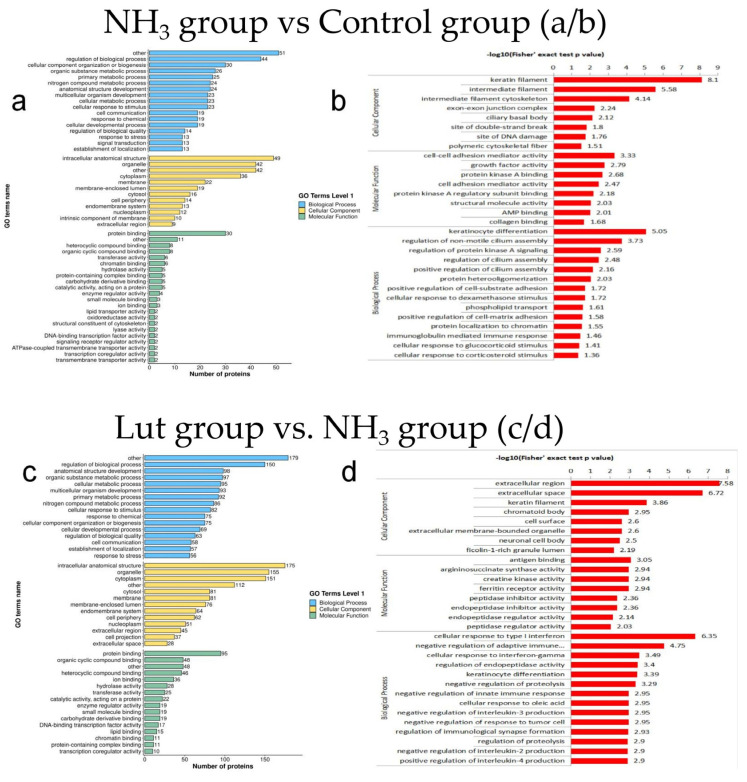

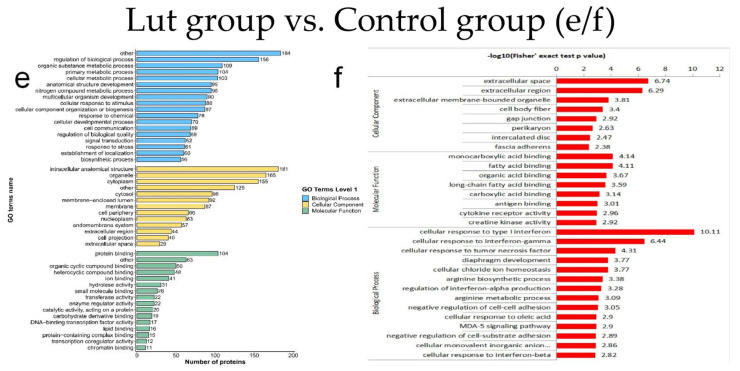
Differential expression protein GO function annotation and enrichment analysis between groups. (**a**) Bar chart of GO classification for DEPs between the NH_3_ group and the control group. (**b**): Bar chart of GO enrichment analysis for DEPs between the NH_3_ group and the control group. (**c**) Bar chart of GO classification for DEPs between the Lut group and the NH_3_ group. (**d**) Bar chart of GO enrichment analysis for DEPs between the Lut group and the NH_3_ group. (**e**) Bar chart of GO classification for DEPs between the Lut group and the control group. (**f**) Bar chart of GO enrichment analysis for DEPs between the Lut group and the control group. Figure 5b,d,f display the top 20 most significantly enriched functions, with the vertical axis representing the corresponding GO functional descriptions and the horizontal axis indicating the −Log10-transformed enrichment significance *p*-values; the larger the value, the stronger the enrichment significance.

The findings of the GO functional classification and enrichment analysis are shown in Figure 5. As illustrated in Figure 5a, the GO secondary classification for DEPs in the NH_3_ vs. Control group yielded a total of 804 GO terms. Among these, 403 terms were associated with biological processes, 117 with molecular functions, and the remaining 284 terms with cellular components. Figure 5b presents the findings of the GO enrichment analysis for DEPs in the NH_3_ vs. Control group. In the category of biological processes, the DEPs were predominantly enriched in processes regulating keratinocyte differentiation and modulating non-motile cilia assembly. Regarding cellular components, the DEPs were primarily localized to keratin filaments and intermediate filaments. In the molecular function classification, the highest proportion of DEPs was involved in regulating cell–cell adhesion and growth factor activities. As depicted in Figure 5c, the GO secondary classification for DEPs in the Lut vs. NH_3_ group identified a total of 3012 GO terms. Within this set, 1425 terms were categorized under biological processes, 469 terms under molecular functions, and the remaining 1118 terms under cellular components. Figure 5d displays the findings of the GO enrichment analysis for DEPs in the Lut vs. NH_3_ group. The DEPs were mainly enriched in functions related to cellular reactions to type I interferons and the inhibition of adaptive immune reactions. In terms of cellular components, the DEPs were primarily found in the extracellular region and the extracellular space. For molecular functions, most of the DEPs were associated with antigenic binding and ferritin receptor activity. Figure 5e illustrates that the GO enrichment analysis for the Lut vs. Control group identified a total of 3352 GO terms. Within this comprehensive set, 1635 terms were associated with biological processes, 517 with molecular functions, and 1200 with cellular components. As depicted in Figure 5f, the DEPs in the Lut vs. Control group were predominantly involved in cellular responses to type I interferons, interferon-gamma, and tumor necrosis factor in terms of biological processes. Regarding cellular components, these proteins were primarily localized to the extracellular space, the extracellular region, and extracellular membrane-bound organelles. Within the domain of molecular functions, the DEPs were notably engaged in the binding of monocarboxylic acids, fatty acids, organic acids, and extended-chain fatty acids.

### 3.6. KEGG Pathway Clustering Analysis

**Figure 6:**
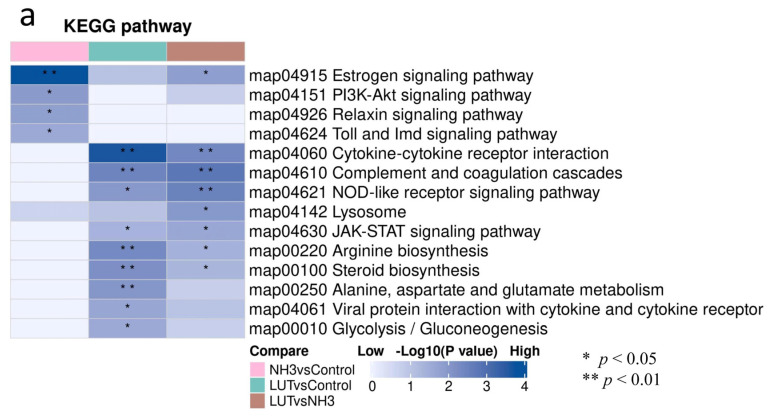

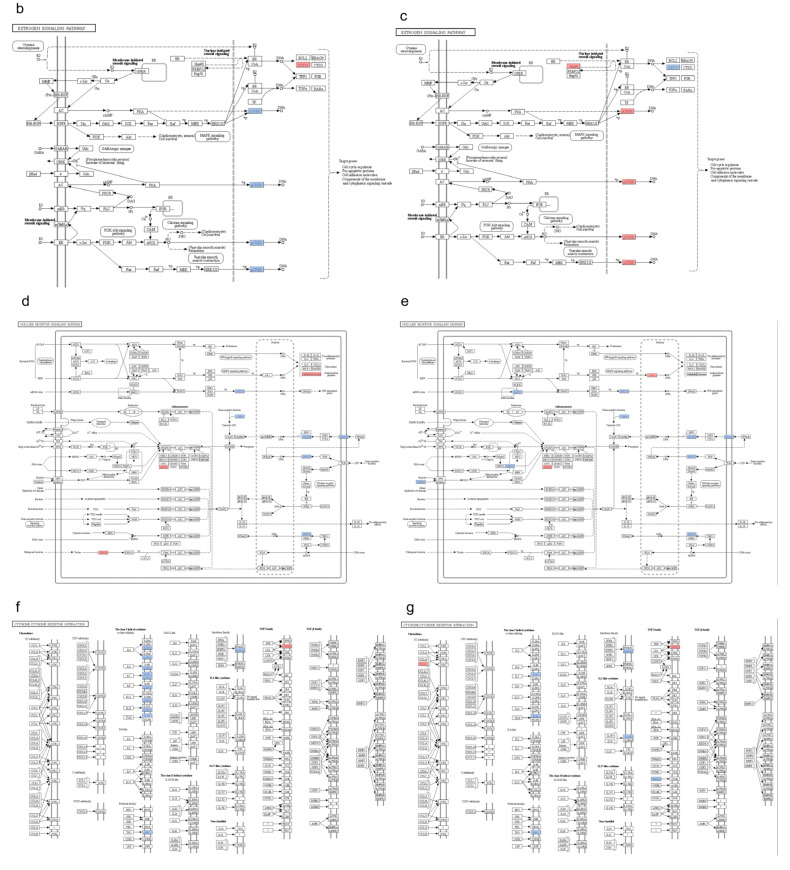

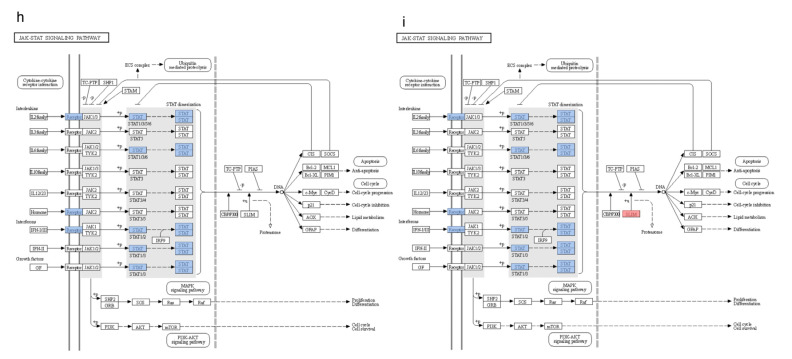
Cluster analysis of differential protein KEGG pathways. (**a**): KEGG clustering analysis diagram. The color blocks corresponding to the functional descriptions enriched by DEPs in different comparison groups indicate the level of enrichment significance. Blue represents high enrichment significance; blue-white represents low enrichment significance; * indicates *p*-value < 0.05; ** indicates *p*-value < 0.01. (**b**) Estrogen signaling pathway diagram for the NH_3_ vs. Control group. (**c**) Estrogen signaling pathway diagram for the Lut vs. NH_3_ group. (**d**) NOD-like receptor signaling pathway diagram for the Lut vs. NH_3_ group. (**e**) NOD-like receptor signaling pathway diagram for the Lut vs. Control group. (**f**) Interaction between cytokines and cytokine receptors diagram for the Lut vs. NH_3_ group. (**g**) Interaction between cytokines and cytokine receptors diagram for the Lut vs. Control group. (**h**) JAK-STAT signaling pathway diagram for the Lut vs. NH_3_ group. (**i**) JAK-STAT signaling pathway diagram for the Lut vs. Control group.

The KEGG pathway mappings for DEPs are illustrated in Figure 6a. In the NH_3_ vs. Control group, these proteins were predominantly associated with the estrogen signaling pathway, the PI3K-Akt signaling pathway, the relaxin signaling pathway, and the Toll and Imd signaling pathways. The receptor signaling pathway; the lysosome signaling pathway; the IL-17 signaling pathway; the synthesis, secretion, and action of parathyroid hormone; cytokine–cytokine receptor interactions; complement and coagulation cascades; and the JAK-STAT signaling pathway were identified, with notable differences in the NOD-like receptor signaling pathway, cytokine–cytokine receptor interactions, and the complement and coagulation cascade signaling pathways. DEPs in the Lut vs. Control group were involved in the estrogen signaling pathway; mitochondrial function; interactions between viral proteins, cytokines, and cytokine receptors; cytokine–cytokine receptor interactions; arginine biosynthesis; steroid biosynthesis; alanine, aspartate, and glutamate metabolism; and gluconeogenesis signaling pathways. Our analysis revealed that the estrogen metabolism pathway was significantly enriched between the NH_3_ vs. Control group and the Lut vs. NH_3_ group. The common pathways between the NH_3_ vs. Lut group and the Lut vs. Control group included cytokine–cytokine receptor interactions, the complement and coagulation cascade signaling pathways, the NOD-like receptor signaling pathway, the JAK-STAT signaling pathway, the arginine biosynthesis pathway, and the steroid biosynthesis pathway. We hypothesized that the therapeutic effects of Lut on ammonia poisoning are closely related to the following pathways: the estrogen signaling pathway (Figure 6b,c), the NOD-like receptor signaling pathway (Figure 6d,e), cytokine–cytokine receptor interactions (Figure 6f,g), and JAK-STAT signaling pathway (Figure 6h,i).

### 3.7. PPI Analysis of DEPs

**Figure 7:**
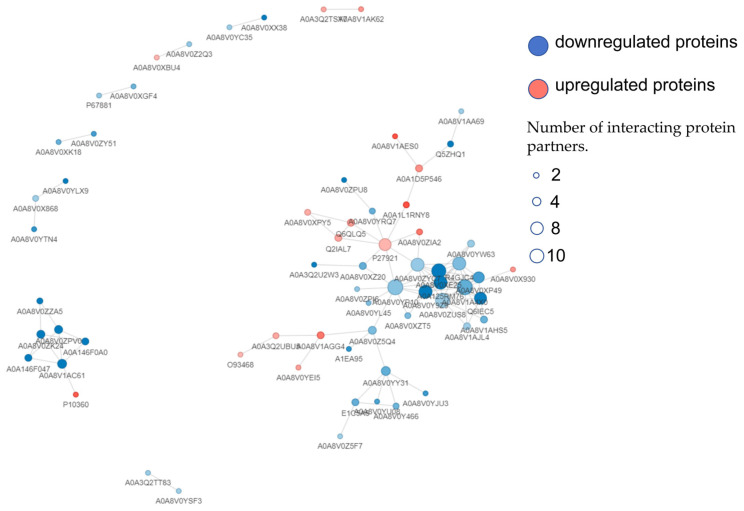
Protein–protein interaction networks. In the diagram, the red filling represents upregulated proteins; the blue filling represents downregulated proteins; circles in the diagram represent DEPs, with different colors indicating the protein’s expression changes (blue for downregulated proteins and red for upregulated proteins); the deeper the color, the greater the fold change, and the size of the circle indicates the number of proteins it interacts with; proteins that are commonly differentially expressed between NH_3_ vs. Control and Lut vs. NH_3_ are marked with red borders in the diagram.

The protein–protein interaction network figure is shown in Figure 7. In the NH_3_ vs. Control group, a total of 69 DEPs were identified, with 14 exhibiting inter connectivity, while the remaining 55 displayed no such associations. In the NH_3_ vs. Lut group, 225 DEPs were detected, of which 73 were found to be interconnected, and 152 lacked any links. Among these 73 interconnected DEPs, the top 10 with the highest degree of connectivity were STAT, a 2′-5′ oligoadenylate synthase, radical S-adenosyl methionine domain-containing 2 (RSAD2), an RNA helicase, a dTMP kinase, IRF7, Interferon-induced protein with tetratricopeptide repeats 5 (IFIT5), ubiquitin carboxyl-terminal hydrolase, Interferon alpha inducible protein 6 (ISG15), and transcription factor JunD. We conducted a comparative analysis of the 73 interconnected DEPs in the NH_3_ vs. Lut group’s PPI network with the 30 overlapping DEPs identified between the NH_3_ vs. Control and NH_3_ vs. Lut groups. This analysis revealed that 10 DEPs were interconnected, indicating potential functional linkages, as illustrated in Figure 7. The 10 DEPs with significant interconnectivity were Keratin 12, Keratin 13, Cathelicidin-1, Keratin 6A, Keratin 7, an IF rod domain-containing protein, Cyclic AMP-dependent transcription factor (ATF-2), Histone H2A, ARF binding protein 3, and Cyclic GMP-AMP synthase.

### 3.8. Lut-Induced Gene Expression Associated with Apoptosis of Chicken Spleen Cells Exposed to Ammonia

**Figure 8:**
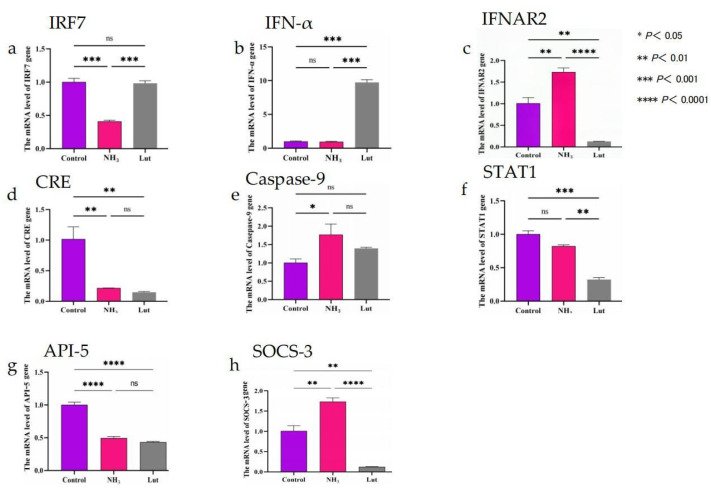
Expression levels of mRNA for apoptosis-related genes. In the figure, the control group is denoted by purple, the NH_3_ group by pink, and the Lut group by gray. Panel (**a**) depicts the mRNA expression levels of IRF7; panel (**b**) shows the mRNA expression levels of IFN-α; panel (**c**) illustrates the mRNA expression levels of IFNAR2; panel (**d**) presents the mRNA expression levels of CRE; panel (**e**) indicates the mRNA expression levels of Caspase-9; panel (**f**) reveals the mRNA expression levels of STAT1; panel (**g**) displays the mRNA expression levels of API-5; and panel (**h**) reflects the mRNA expression levels of SOCS-3.

In this study, chick spleen cells were treated with an ammonia-containing medium. The mRNA expression levels of apoptosis-related genes were measured using qRT-PCR. This method was used to investigate how Lut affects the expression of these genes. The results, shown in Figure 8, indicated that the expression of the IRF-7 gene was significantly higher in both the control and Lut groups than in the NH_3_ group (*p* < 0.001). Additionally, the mRNA level of the IFN-α gene was significantly higher in the Lut group compared to the control and NH_3_ groups (*p* < 0.001). Although the mRNA levels of caspase-9 were lower than those in the NH_3_ group and higher than those in the control group, these differences were not statistically significant (*p* > 0.05). In contrast, the mRNA levels of the API-5 gene were significantly lower in both the NH_3_ and Lut groups compared to the control group (*p* < 0.0001). Additionally, the mRNA level of the SOCS-3 gene was significantly lower in the control group than in the NH_3_ group (*p* < 0.01), while it was significantly reduced in the Lut group compared to both the NH_3_ group (*p* < 0.0001) and the control group (*p* < 0.01). Overall, Lut significantly modulated the expression of apoptosis-related genes (IRF-7, IFN-α, API-5, and SOCS-3) in ammonia-exposed chick blastocysts, whereas caspase-9 showed no statistically significant differences between groups.

## 4. Discussion

Ammonia, a harmful atmospheric gas to human health, also significantly threatens the welfare of poultry and the health of workers in concentrated poultry farming facilities [18]. Research has shown that ammonia can induce renal and intestinal inflammation and damage in broiler chickens [19,20], as well as cellular apoptosis and pyroptosis in splenic cells [4,21,22]. Plant-derived extracts rich in flavonoids, such as Lut, have been reported to reduce oxidative stress and inflammatory responses [23]. Lut can effectively alleviate inflammatory responses [10] and protect against NLRP3-mediated inflammatory diseases [11]. In protecting against chicken ammonia poisoning, Lut not only reduces autophagy and apoptosis of lymphocytes after ammonia exposure [12] but also alleviates the levels of inflammation and pyroptosis [13]. In this study, we conducted a 4D-FastDIA-based quantitative proteomics analysis. GO analysis revealed significant enrichment of DEPs in keratin filament structure and keratinocyte differentiation processes. KEGG clustering analysis showed significant enrichment of DEPs in the estrogen signaling pathway, the NOD-like receptor signaling pathway, cytokine–cytokine receptor interaction, and the JAK-STAT signaling pathway. PPI analysis using the STRING database (confidence score threshold > 0.7) revealed a tightly interconnected network comprising 10 DEPs predominantly localized at the network core with peripheral extensions. Notably, these predicted associations are generated by computational inference algorithms that integrate known interaction data (e.g., co-expression, text mining, and pathway co-occurrence), serving as mechanistic hypotheses for functional linkages rather than direct evidence of physical binding [24]. While such bioinformatic predictions provide valuable insights into pathway crosstalk, orthogonal experimental approaches (e.g., co-immunoprecipitation or affinity purification mass spectrometry) are required to empirically validate these interactions. These results suggest that the significantly enriched pathways may be associated with Lut’s therapeutic effects on ammonia poisoning.

The estrogen signaling pathway, composed of estrogen and estrogen receptors, is the primary route through which estrogen exerts its physiological functions [25]. Estrogen is a pleiotropic hormone with multiple effects [26]. Studies have shown that estrogen can effectively alleviate inflammatory responses by regulating the activation of NLRP3 [27]. Additionally, estrogen might impact the onset and progression of hepatocellular carcinoma through the regulation of inflammation and immune cell infiltration [28]. This study found that in the NH_3_ vs. Control group, four type I keratins in the estrogen signaling pathway showed an upregulated trend; the downregulated protein was the ATF-2. The downregulation of ATF-2 in the estrogen pathway corresponded to the PI3K-Akt signaling pathway, inhibiting cell survival pathways. In contrast, in the Lut vs. NH_3_ group’s estrogen signaling pathway, the regulation of DEPs was opposite to that of the NH_3_ vs. Control group, with upregulated DEPs including ATF-2 and Heat Shock Protein 90 alpha (HSP90α); the downregulated DEPs were four keratins. The upregulation of ATF-2 and HSP90α corresponded to the PI3K-Akt signaling pathway, activating cell survival and promoting AKT phosphorylation—a mechanism consistent with Lut’s role in enhancing PI3K-Akt activity, as observed in splenic lymphocytes under stress conditions [29]. qRT-PCR assays showed a significant reduction in CRE mRNA in the NH_3_ and Lut groups versus controls (*p* < 0.01), though Lut’s lower CRE mRNA than NH_3_ was not statistically significant. The discrepancies between proteomics and qRT-PCR findings may arise from a multilayered regulatory network encompassing both molecular mechanisms and technical variables. At the molecular level, three interconnected factors predominate: (1) mRNA–protein temporal decoupling manifested through CRE mRNA’s intrinsic instability (accounting for qRT-PCR’s downward trend) versus sustained protein persistence from pre-existing pools and delayed degradation [30]; (2) dynamic transcriptional control via negative feedback loops that suppress CRE expression when protein concentrations reach threshold levels, a regulatory mechanism commonly observed in cellular homeostasis [31]; (3) translational efficiency disparities among alternatively spliced variants, particularly given that qRT-PCR targeted specific isoforms while other high-efficiency variants might disproportionately contribute to protein synthesis. Beyond these biological determinants, technical considerations including post-translational modification detection limitations in proteomics, temporal mismatches in sample collection between transcriptomic/proteomic profiling, and methodological variances in both platforms (e.g., primer specificity in qRT-PCR versus peptide identification thresholds in mass spectrometry) likely compound the observed discordances. Notably, proteomic evidence positions Lut’s ammonia-protective effects within the estrogen signaling axis, suggesting that pathway-specific post-transcriptional regulation mechanisms may particularly underlie this pharmacological phenomenon.

The NOD-like receptor signaling pathway plays a crucial role in mediating immune responses such as stress and inflammation-induced tissue damage [32]. Furthermore, it plays a notable role in the development of gastrointestinal inflammatory conditions and cancers [33]. Current research indicates that the NLRP3 inflammasome can control the production of pro-inflammatory cytokines Interleukin-18 (IL-18) and Interleukin-1 beta (IL-1β) through caspase-1 activation [34] and induce pyroptotic cell death [35]. In the Lut vs. NH_3_ group, 10 DEPs are associated with the NOD-like receptor signaling pathway, among which the downregulated proteins include IFNAR2, STAT, IFR-7, guanylate-binding protein (GBP), and proteins containing the GB1/RHD3-type G domain. The expression of these downregulated proteins may suppress the function of the Gasdermin D (GSDMD) protein. GSDMD is a key pro-death executor protein involved in inflammatory signal transduction, the activation of various inflammasomes, and the release of downstream inflammatory cytokines [36]. By downregulating protein expression, the activity of GSDMD protein is inhibited. This predicted result is consistent with the existing literature [37]. In the NOD-like receptor signaling pathway, Lut inhibited the expression of the NLRP3 inflammasome by reducing the expression levels of the mitochondrial antiviral signaling protein and nicotinamide phosphoribosyltransferase. Previous studies have shown that IFN-α can induce apoptosis [38]. Lut reduced NLRP3 inflammasome expression by decreasing mitochondrial antiviral signaling protein and nicotinamide phosphoribosyltransferase levels. Lut also inhibited IFN-α expression by downregulating the mitochondrial antiviral signaling protein and IRF-3, reducing apoptosis rates. Furthermore, Lut inhibited GSDMD function by downregulating IFNAR2, STAT, IRF-3, IRF-7, and GBP. These findings suggest that Lut may interfere with ammonia-induced inflammation and apoptosis through the NOD-like receptor signaling pathway. This study also found that Lut significantly increased IRF-7 and IFN-α mRNA levels but decreased IFNAR2 and STAT1 mRNA levels compared to the NH_3_ and control groups. This pattern aligns with proteomics data, further supporting the hypothesis that Lut may influence ammonia-induced apoptosis via this pathway.

The cytokine–cytokine receptor interaction pathway plays a key role in innate and adaptive inflammatory host defense and cell death processes [39,40,41]. In the comparison between the Lut and NH_3_ groups, five DEPs were identified as being associated with the cytokine–cytokine receptor interaction signaling pathway. Among these, Tumor Necrosis Factor Receptor Superfamily Member 1B was notably upregulated, while four proteins were downregulated, comprising IL-7 Receptor Subunit Alpha (IL-7Rα), IFNAR2, IL-2 Receptor Subunit Gamma (IL-2Rγ), and the thrombopoietin receptor. In the Lut vs. Control group, the cytokine–cytokine receptor interaction pathway exhibited the upregulation of Tumor Necrosis Factor Receptor Superfamily Member 1B and C-C motif chemokine 21, which contrasted with the downregulation of Tumor Necrosis Factor Ligand Superfamily Member 5, IL-18 Receptor 1 (IL-18R1), IL-7Rα, IFNAR2, and the thrombopoietin receptor. The downregulation of the aforementioned proteins curtailed the propagation of downstream inflammatory responses within this pathway. Specifically, the reduced expression levels of the IL-7 receptor, the thrombopoietin receptor, and interferon receptor 2 resulted in a diminished number of receptors available for the JAK-STAT signaling cascade, thereby impeding the generation of further inflammatory reactions. The downregulation of IFNAR2 and IL-7Rα aligns with the negative feedback mechanism [42], in which PKA-dependent activation of SHP2 phosphatase dephosphorylates JAK1, thereby suppressing STAT1/3 phosphorylation and downstream Nuclear Factor-kappa B (NF-κB) activation. This reduces the availability of JAK-STAT signaling components, hindering inflammatory cascades. The mRNA levels of the IFN-α gene in the Lut group were significantly elevated compared to the control and NH_3_ groups (*p* < 0.001). However, IFN-α was not detected as a differentially expressed protein in the proteomics analysis. To further investigate this inconsistency, we conducted a detailed analysis from multiple perspectives. We suggest that this discrepancy may be due to the combined effects of several factors: mRNA stability, post-translational modifications, differences in sample processing, post-transcriptional regulation, negative feedback mechanisms, experimental errors, and time-delay effects. Additionally, we observed a downward trend in the mRNA expression levels of IFNAR2, which was consistent with the downregulation observed in the proteomics analysis. By combining the proteomics and qRT-PCR results for IFNAR2, we hypothesize that Lut protects cells from apoptosis induced by ammonia poisoning by reducing the gene and protein expression levels of IFNAR2. This finding supports our hypothesis that Lut can reduce cell apoptosis and protect against ammonia poisoning by modulating the cytokine–cytokine receptor interaction signaling pathway. This regulatory effect of Lut is instrumental in safeguarding chickens against the detrimental effects of ammonia poisoning. However, the clinical relevance of these mechanisms requires validation in avian-specific models, given the known species differences in JAK-STAT pathway regulation compared to mammalian systems [42].

The JAK-STAT signaling pathway plays a crucial regulatory role in cell growth, proliferation, differentiation, and apoptosis [43,44]. This study identified five DEPs associated with the JAK-STAT signaling pathway in the Lut vs. NH_3_ group. These DEPs include the IL-7Rα, IFNAR2, STAT proteins, IL-2Rγ, and TPOR, all of which exhibited a downregulated trend. To elucidate the protective mechanism of Lut in ameliorating ammonia poisoning, we conducted an analysis of the JAK-STAT signaling pathway in the Lut vs. Control group. In the Lut vs. Control group, the JAK-STAT signaling pathway revealed downregulated DEPs that were consistent with those observed in the Lut vs. NH_3_ group. The qRT-PCR results indicated that the mRNA levels of IFNAR2 and STAT1 were trending downward. This observation was in strong agreement with the downregulation noted in the proteomics analysis. It is hypothesized that Lut might reduce the expression of IFNAR2 and STAT1 at both the transcriptional and translational levels by influencing the JAK-STAT signaling pathway, thus preventing the initiation of downstream apoptotic processes. This conjecture is corroborated by the findings reported in references [45,46], supporting the idea that Lut could modulate this pathway to inhibit downstream apoptotic processes. However, further research is needed to confirm these effects and explore the specific mechanisms involved.

## 5. Conclusions

To encapsulate, our investigation suggests that Lut may mitigate inflammatory reactions induced by ammonia inhalation through the modulation of key signaling cascades, including the estrogen signaling pathway, the NOD-like receptor signaling pathway, the cytokine–cytokine receptor interaction pathway, and the JAK-STAT signaling pathway. While proteomic and transcriptomic data confirm Lut’s immunomodulatory effects, the mismatch between IFN-α mRNA and protein levels suggests post-transcriptional regulation, potentially due to species-specific feedback mechanisms or ubiquitination-mediated degradation. Lut emerges as a multi-target phytochemical for ammonia detoxification, with potential applications in poultry health management through biomarker monitoring and nutraceutical formulations. However, the validation of these effects in avian models and dose optimization are essential for clinical translation, given the mechanistic divergences from mammalian systems.

## Figures and Tables

**Table 1:** Primers of genes.

Gene	Primer (5′-3′)	Size (bp)
β-actin	F: CCCAGCCATGTATGTAGCCATCC R: AACACCATCACCAGAGTCCATCAC	91
IRF7	F: CAGCACAAAGCCCAAGGAGTC R: TTGCCACTGTTGAGGGAGGAG	114
IFN-α	F: ACCACCCACGACATCCTTCAG R: GGCTTTGGCGTTGGCTGTC	88
IFNAR2	F: TTCCCAAGAAGATGCTGTTGACTG R: TGTGGTTTCTGCGTGCTTTCTG	87
CRE	F: GTGGCGATGGATGCGTTGTC R: CTGCTGTTGCTGGGAAGTTGTC	81
Caspase-9	F: AAGGTGAGTGGCTCGTGGTAC R: GTAGCATGGTTAGCAGGTCTTCAG	85
STAT1	F: CTCTGGAACGATGGCTGTATCATG R: GCTCCCTCTTTACTGCTTTCACTG	119
API-5	F: TGCAGTTCAGTTATGTCGAGTGTC R: TTTGGCTGTGAGGAAATCTGGAAG	80
SOCS-3	F: GCCTCAAGACGTTCAGCTCTAAG R: CTCCAGTAGAAGCCGCTCTCC	85

This table presents the primers and their related information used for gene expression detection. The columns are as follows: “Gene” indicates the name of the gene; “Primer (5′-3′)” shows the primer sequence, with “F” representing the forward primer for amplifying the 5′ end of the target gene and “R” representing the reverse primer for amplifying the 3′ end; “Size (bp)” denotes the length of the amplified fragment in base pairs. All primers have been validated for use in qPCR analysis.

## Data Availability

All relevant data are within the paper. The data used to support the findings of this study are available upon reasonable request.

## Abbreviations

The following abbreviations are used in this manuscript:

NH_3_	Ammonia
Lut	Luteolin
4D-FastDIA	Four-Dimensional Fast Data-Independent Acquisition
DEPs	Differentially Expressed Proteins
GO	Gene Ontology
KEGG	Kyoto Encyclopedia of Genes and Genomes
qRT-PCR	quantitative real-time polymerase chain reaction
IFNAR2	Interferon Alpha and Beta Receptor subunit 2
STAT1	Signal Transducer and Activator of Transcription 1
HPLC	High-Performance Liquid Chromatography
MS	Mass Spectrometry
DIA	Data-Independent Acquisition
FDR	False Discovery Rate
COGs	Clusters of Orthologous Groups
PPI	Protein–Protein Interaction
IRF-7	Interferon Regulatory Factor 7
IFN-α	Interferon-alpha
CRE	Cyclic AMP-responsive Element
Caspase-9	Cysteinyl Aspartate Specific Protease 9
API-5	Apoptosis Inhibitor 5
SOCS-3	Suppressor of Cytokine Signaling 3
IL	Interleukin
GSDMD	Gasdermin D
NF-κB	Nuclear Factor-kappa B
